# Nuclear exclusion of YAP exacerbates podocyte apoptosis and disease progression in Adriamycin-induced focal segmental glomerulosclerosis

**DOI:** 10.1038/s41374-020-00503-3

**Published:** 2020-11-17

**Authors:** Qiyuan Zhuang, Fang Li, Jun Liu, Hongyu Wang, Yuchen Tian, Zhigang Zhang, Feng Wang, Zhonghua Zhao, Jianchun Chen, Huijuan Wu

**Affiliations:** 1 Department of Pathology, School of Basic Medical Sciences, Fudan University, Shanghai, China; 2 Department of Neurosurgery, Huashan Hospital, Fudan University, Shanghai, China; 3 Department of Nephrology, Zhongshan Hospital, Fudan University, Shanghai, China; 4 Department of Nephrology, Shanghai General Hospital, Shanghai Jiao Tong University School of Medicine, Shanghai, China; 5 Department of Nephrology, Shanghai Jiao Tong University Affiliated Sixth People’s Hospital, Shanghai, China; 6 Division of Nephrology in Department of Medicine, Vanderbilt University School of Medicine, Nashville, TN USA

**Keywords:** Focal segmental glomerulosclerosis, Apoptosis

## Abstract

Focal segmental glomerulosclerosis (FSGS) is a chronic glomerular disease with poor clinical outcomes. Podocyte loss via apoptosis is one important mechanism underlying the pathogenesis of FSGS. Recently, Yes-associated-protein (YAP), a key downstream protein in the Hippo pathway, was identified as an activator for multiple gene transcriptional factors in the nucleus to control cell proliferation and apoptosis. To investigate the potential role of YAP in the progression of FSGS, we examined kidney samples from patients with minimal change disease or FSGS and found that increases in podocyte apoptosis is positively correlated with the cytoplasmic distribution of YAP in human FSGS. Utilizing an established mT/mG transgenic mouse model and primary cultured podocytes, we found that YAP was distributed uniformly in nucleus and cytoplasm in the podocytes of control animals. Adriamycin treatment induced gradual nuclear exclusion of YAP with enhanced phospho-YAP/YAP ratio, accompanied by the induction of podocyte apoptosis both in vivo and in vitro. Moreover, we used verteporfin to treat an Adriamycin-induced FSGS mouse model, and found YAP inhibition by verteporfin induced nuclear exclusion of YAP, thus increasing podocyte apoptosis and accelerating disease progression. Therefore, our findings suggest that YAP nuclear distribution and activation in podocytes is an important endogenous anti-apoptotic mechanism during the progression of FSGS.

## Abstract (web summary)

An increase in podocyte apoptosis is positively correlated with the cytoplasmic distribution of YAP in focal segmental glomerulosclerosis (FSGS). YAP inhibition by verteporfin induces nuclear exclusion of YAP, thus increasing the podocyte apoptosis and accelerating disease progression. Therefore, this study suggests that YAP activation and nuclear distribution in podocytes is an endogenous anti-apoptotic mechanism during the progression of FSGS.

## Introduction

The main manifestation of focal segmental glomerulosclerosis (FSGS) is nephrotic syndrome, a common chronic glomerular disease with poor clinical outcomes [1]. Most of FSGS patients are cortisone-resistant, with a tendency to progress to end-stage renal disease [2]. Podocyte injury (including cytoskeleton impairment, hypertrophy and autophagy) and podocyte loss (including apoptosis and detachment), as well as exposure of glomeruli basement membrane, are critical mechanisms leading to the pathogenesis and progression of FSGS [3–5]. In diphtheria toxin-induced mouse models, FSGS was observed histologically when the percentage of podocytes loss exceeded 20%. Also, the degree of podocyte loss paralleled with the severity of proteinuria and the extent of renal function decline [6]. Since podocytes are highly differentiated and mature, preventing podocytes from injury and loss is of importance in order to slow or antagonize the progression of FSGS.

The Hippo pathway is an important signaling pathway regulating cell proliferation, differentiation and apoptosis. Yes-associated-protein (YAP), one key downstream effector of Hippo pathway, was recognized as an activator for multiple gene transcriptional factors in nucleus, where they bind to and activate TEAD family transcription factors, by its dephosphorylated form [7, 8]. The phosphorylated YAP is retained in the cytoplasm by binding to 14-3-3, followed by degradation through ubiquitin-proteasome system [9], or autophagy upon lysosomal pathway [10].

Although dysregulation of Hippo pathway is closely correlated with prognosis and the survival rate in various human cancers [11–14], its role in renal diseases need to be further investigated. A recent study showed that *YAP* deletion or mutation in cap mesenchyme resulted in impairment of nephron formation and morphogenesis during renal development [15]. Selectively deleted YAP in podocytes led to podocyte depletion, proteinuria, and increase of serum creatinine, which histologically featured characteristic of FSGS [16]. Besides, other studies showed that mutation of RhoA, a protein highly related to cytoskeleton, and Cdc42 can induce apoptosis of podocytes in vitro via inhibiting Hippo pathway [17]. These results indicate that YAP, a key downstream protein in the Hippo pathway, is important for podocyte injury in the progression of FSGS.

The current study examined the relationship between YAP localization and phosphorylation with podocyte apoptosis in kidney samples from patients with FSGS and an Adriamycin-induced FSGS mouse model, and primary cultured podocytes as well. In addition, we observed the effect of verteporfin, a YAP inhibitor, on YAP nuclear exclusion, podocyte apoptosis and the progression of FSGS. We found that YAP might be a potential therapeutic target for FSGS.

## Materials and methods

### Generation and genotyping of *NPHS2-Cre*; *mT/mG* mice

*NPHS2-Cre*; *mT/mG* mice were generated by crossing *NPHS2-Cre* mice (The Jackson laboratory, 008205) with *Gt(ROSA)26Sor*^*tm4(ACTB-tdTomato,EGFP)Luo*^ (The Jackson laboratory, 007676). Genotyping was performed according to previously described protocols [18]. Male F1 mice were used for experiment. PCR primers used for *NPHS2-Cre* included: 5′-GCGCTGCTGCTCCAG-3′ and 5′-CGGTTATTCAACTTGCACCA-3′, while 5′-CTTTAAGCCTGCCCAGAAGA-3′, 5′-AGGGAGCTGCAGTGGAGTAG-3’ and 5′-TAGAGCTTGCGGAACCCTTC-3′ were used to detect *mT/mG*. PCR conditions for *mT/mG* are: 94 °C for 3 min followed by 94 °C for 30 s, 58 °C for 1 min, and 72 °C for 60 s for 30 cycles, with an additional 2-min extension at 72 °C. For NPHS2-Cre: 94 °C for 3 min followed by 94 °C for 30 s, 51.7 °C for 1 min, and 72 °C for 60 s for 35 cycles, with an additional 2-min extension at 72 °C.

### Administration of Adriamycin and verteporfin to mT/mG mice

Adriamycin was purchased from Sigma, USA, and was administrated in mT/mG mice at several different doses (15, 17, 19, 22 mg/kg body wt) by intravenous injection (one dose at 5 weeks of age). Verteporfin was purchased from TargetMol, USA and was administrated in mT/mG mice at 100 mg/kg body wt by intraperitoneal injection every other day for 1, 2, or 3 weeks (starting from the 1st day of the Adriamycin-induced nephropathy mouse model construction). verteporfin dosages are consistent with a previous report [19, 20]. The mice were then sacrificed.

### Kidney histology, immunohistochemistry, immunofluorescence, immunoblotting and electron microscopic imaging of kidney tissue

Mouse kidney was fixed by 4% paraformaldehyde for paraffin-embedded kidney sections (5 μm), which follows staining techniques after deparaffinized and rehydrated. For kidney histologic examination, H&E staining was performed using the standard methods [21]. Immunohistochemistry and immunofluorescence staining were performed as described previously [22]. In brief, endogenous peroxidase was removed using 3% H_2_O_2_, and the antigen was retrieved in citrate buffer, following with block with 5% normal sheep serum and incubation with primary antibody at 4 °C overnight (YAP: 1:100; nephrin: 1:100; p-YAP (S127):1:50; p-YAP (S397): 1:50; activated Caspase 3: 1:100; podocin: 1:100). After washed three times in PBS, the appropriate secondary antibodies were applied for 1 h in room temperature. For immunohistochemistry, the signals were visualized using liquid DAB + substrate chromogen system (Dako, USA), followed by counterstaining with hematoxylin and capturing images using the Vectra Camera System. For immunofluorescence staining, after incubation with the primary antibodies indicated and washing with PBS, the sections were incubated with Alexa Fluor^TM^ 549-conjugated secondary antibodies and Dylight 488-conjugated secondary antibodies for 1 h, and images were captured using the Zeiss camera. Immunoblotting and analyses were performed as previously reported [23]. The preparation and examination of Electron microscopic grids was performed by the Department of Pathology, School of Basic Medical Sciences, Fudan University.

### Podocyte number, glomerular volume and podocyte density

Section pairs were cut into 2 µm and collected every 10 µm from the glomerulus by using a diamond knife. Twenty pairs of sections were saved per kidney. With the assumption that per podocyte has only 1 nucleus, podocyte nuclei were used as surrogates for podocytes. We used the dissector/fractionator principle for measurement of podocyte number [24], which, in brief, counts the unique nuclei only present in the first section of each dissector pair but not in the second. The Cavalieri principle was also used to measure glomerular volume [24]. Podocyte density was calculated as dividing podocyte number by glomerular volume, expressing as number per cubic micrometer [25].

### Podocyte nuclear YAP localization

After triple immunofluorescence staining with nephrin, YAP and DAPI, podocytes were firstly confirmed by nephrin positive staining, and then the area of YAP and DAPI double-positive staining that surrounded by red nephrin staining (the area of YAP in podocyte nuclear) were counted for the numerator, which was divided by the sum of the area with DAPI that also surrounded by red nephrin staining (all the area of podocyte nuclear). The positive area was calculated by Image J [26], and at least 40 images were captured from each group (*n* ≥ 5 cases per group, 8 images per case).

### Antibodies

Antibodies used in this study included YAP, p-YAP (S127), p-YAP (S397), and Caspase 3 were purchased from Cell Signaling Technology. Antibodies against β-actin and nephrin were purchased from R&D Systems. Peroxidase conjugated goat anti-mice IgG and goat anti-rabbit IgG antibody were purchased from ProteinTech Group.

### Measurement of Kidney Function and TUNEL Staining

Serum creatinine and BUN was measured in duplicate using commercial kits according to the manufacturer’s protocol (Jiancheng Nanjing, China). For TUNEL labeling, mice kidney sections were fixed by 4% methanol-free formaldehyde and then were washed once with PBS, following by permeabilization with proteinase K for 10 min at room temperature. After pre-equilibrate, incubation buffer containing equilibration buffer, nucleotide mix and rTdT enzyme (Promega, USA) were added at 37 °C for 1 h. Subsequently, after three times of wash with PBS for 5 min, DAPI (Life Technology) nuclear stain was added in mounting medium and proceed to analysis according to the manufacturer’s protocol.

### Primary podocytes culture and treatment

The glomerular cells were obtained as previously described [27], briefly, glomerular cells was collected from male *NPHS2-Cre*; *mT/mG* mice at 5 weeks of age, by sieving through 100 μm filters and then 40 μm filters, following plated in type I collagen-coated dishes, cultured in DMEM/Low Glucose with 10% FBS, Insulin, Transferrin, Sodium Selenite Media Supplement, ITS (Sigma-Aldrich), Hydrocortisone, Na-3,3′, 5-Ttiiodo-L-Thyronine,100 U/ml penicillin, and 100 μg/ml streptomycin. The glomerular cells were sorted by flow cytometry and the primary podocytes were cultured at 37 °C with 95% air and 5% CO_2_ for at least 15 days. Primary podocytes were then exposed to Adriamycin at 0.25 μg/ml for 0, 12, 24, or 48 h. 250 nmol/L verteporfin or vehicle was applied to inhibit YAP for 0, 12, or 48 h.

### Statistical analysis

The experiments were all repeated for at least three times in independent biological replicates. Data are presented as means ± SD. One-way analysis of variance was used to assess the differences between multiple groups, and Least Significant Difference (LSD) was used as the post-test to analyze difference between any two mean, and *P* < 0.05 was considered significant.

### Study approval

Permission on performing animal experiment for research purposes (No. 20170223-046) and the use of human tissue (No. 2017-C008) was approved by the Ethics Committee of School of Basic Medical Sciences, Fudan University, China. All procedures were carried out according to the approved guidelines.

## Results

### The localization of YAP with podocyte apoptosis in cases of minimal change disease (MCD) or FSGS

**Fig. 1:**
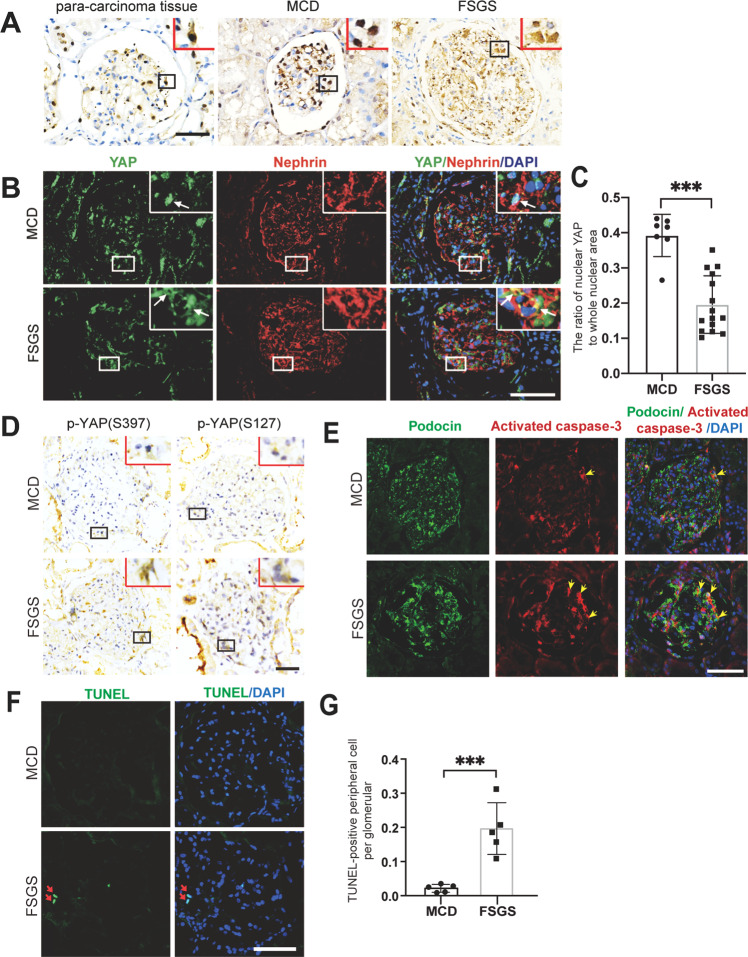
Expression and phosphorylation of YAP and apoptosis of podocytes in cases of MCD or FSGS. **A** Shown are representative immunohistochemical staining images from para-carcinoma tissue (*n* = 5), minimal change disease (MCD, *n* = 5) and FSGS (*n* = 5) cases. **B** Double immunofluorescence staining with YAP and nephrin in cases of minimal change disease (MCD, *n* = 7) and FSGS cases (*n* = 15). Arrows point out nucleus YAP in MCD and cytoplasm YAP in FSGS. **C** YAP positive nuclear area was calculated from eight randomly captured images at each case (case number: *n* = 7 in MCD, *n* = 15 in FSGS). Only area of YAP and DAPI double-positive staining that surrounded by red nephrin staining (indicate the YAP in podocyte nuclear) were counted for the numerator, which was divided by the sum of the area with DAPI that also surrounded by red nephrin staining (indicate all the area of podocyte nuclear). **D** Immunohistochemical staining showed more p-YAP (S397) and p-YAP (S127) in glomeruli of FSGS (*n* = 15) than in MCD (*n* = 7). **E** Double immunofluorescence staining with activated Caspase 3 and podocyte cytoplasm marker podocin. Representative apoptotic podocytes were marked with arrow (yellow). **F** Fluorometric TUNEL system were used to label apoptotic cell in glomeruli and DAPI to label nuclei showed more apoptotic cells in peripheral area of glomeruli in cases of FSGS than in MCD. **G** Apoptotic cells in peripheral area of glomeruli were calculated from 25 randomly captured images at each group (*n* = 5 cases per group, 5 images per case). Only TUNEL-positive nuclei in peripheral area of glomeruli was counted for the numerator, which was divided by the sum of DAPI-positive nuclei and TUNEL-positive nuclei within glomeruli. Data are expressed as means ± SEM. ***P < 0.001 (Scale bar: 30 μm in **A**, **B**, **E**, **B**; 20 μm in **B**).

Mass et al. recently proposed that MCD is the early stage of FSGS [28]. We thus examined the expression of YAP in the glomeruli of normal renal tissue adjacent to cancer and in the glomeruli of MCD and FSGS (not otherwise specified, NOS) by immunohistochemical staining. The results show that YAP is more evenly distributed in cytoplasm and nuclei of the cells around the glomeruli (mainly podocytes) of normal renal tissue; in MCD, YAP is mainly expressed in the nucleus of podocytes; while in FSGS, YAP is abundantly expressed and accumulated in the cytoplasm of podocytes (Fig. 1A; Supplementary Fig. 3A). Double immunofluorescence staining with YAP and nephrin (a podocyte marker) further confirmed that YAP is mainly located in podocyte nuclei in MCD patients, but in podocyte cytoplasm in FSGS patients (Fig. 1B, C). Since cytoplasmic YAP is the phosphorylated form and nuclear YAP is the non-phosphorylated form [29], we thus examined p-YAP (S397) and p-YAP (S127), the important phosphorylation sites of YAP [30], and showed expression of both p-YAP (S397) and p-YAP (S127) in glomeruli were higher in FSGS than that in MCD (Fig. 1D; Supplementary Fig. 3B).

Since YAP is an anti-apoptotic protein that binds to the TEAD transcription factor to decrease cell death, we then examined podocyte apoptosis in MCD and FSGS. TUNEL staining and quantification of TUNEL-positive cells in peripheral area of glomeruli, as well as the double-staining of activated Caspase 3 and podocyte cytoplasm marker podocin, showed more apoptotic podocytes in FSGS patients compared to MCD patients (Fig. 1E–G). These results indicate that increases in podocyte apoptosis is positively related to cytoplasmic distribution of YAP in human FSGS.

### Adriamycin induced gradual nuclear exclusion of YAP accompanied with the induction of podocyte apoptosis in a FSGS mouse model

**Fig. 2:**
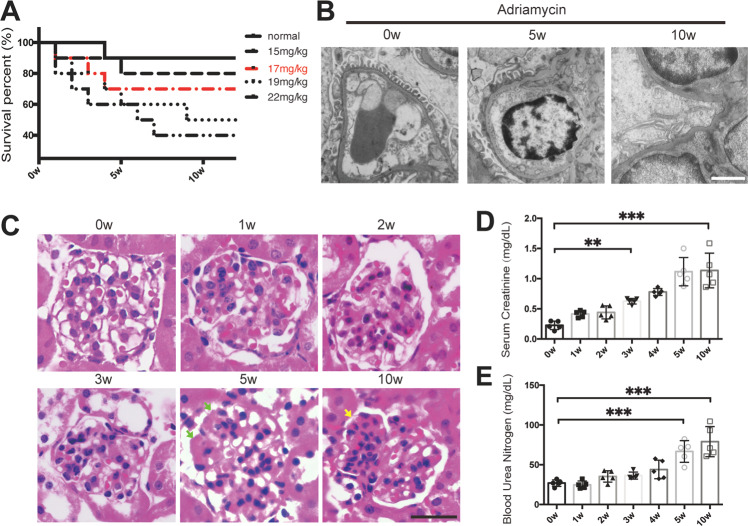
Characterization of the Adriamycin-induced FSGS mouse model. **A** Five-week-old mice were subjected to Adriamycin injections with concentration of 15,17,19,22 mg/kg bwt for single dose, in which 17 mg/kg bwt was chosen for its effects and relatively high survival rate. **B** Shown are representative electron microscopy images from mice sacrificed at 0, 5, 10 weeks after Adriamycin or vehicle treatment. **C** Representative kidney sections with H&E staining from mice sacrificed at 0, 1, 2, 3, 5, and 10 weeks after Adriamycin treatment, with the point of 5 week showing increased matrix in mesangial area (green arrows) and 10 week showing typical segmental sclerosis lesions (yellow arrow). **D**, **E** Statistically significant increases in serum creatinine, blood urea nitrogen (BUN) in mice treated with Adriamycin indicate deteriorating kidney function. Data are expressed as means ± SEM. **P < 0.01, ***P < 0.001 compared with control; *n* = 5 mice per group (Scale bars: 3 μm in **B**; 25 μm in **C**).

To investigate the relationship between YAP nuclear exclusion and podocyte apoptosis in the progression of FSGS, an Adriamycin-induced FSGS mouse model was constructed (Fig. 2A) by using *mT/mG*; *NPHS2-Cre* mice, which were generated as illustrated in the “Materials and methods” (We used 17 mg Adriamycin/kg body weight for its safety and efficiency; see Supplementary Fig. 1A, B for more about creation of the model). Electron microscopy showed that podocyte foot processes were extensively fused and formed numerous microvilli at the 5th week; and foot processes largely disappeared at the 10th week of FSGS mouse model (Fig. 2, B). H&E staining showed that the extracellular matrix in mesangial area increased at the 5th week, and segmental sclerosis was easily seen at the 10th week (Fig. 2C). Serum creatinine increased from the 3rd week and BUN increased from the 5th week, which was more striking at 10th week after Adriamycin treatment (creatinine from 0.254 ± 0.105 mg/dL to 1.18 ± 0.035 mg/dL, Fig. 2D; BUN from 26.4 ± 3.6 mg/dL to 80.6 ± 2.5 mg/dL, Fig. 2E), suggesting a gradually renal function deterioration. Therefore, the typical characteristics of FSGS was observed at the time point of the 10th week after Adriamycin treatment.

**Fig. 3:**
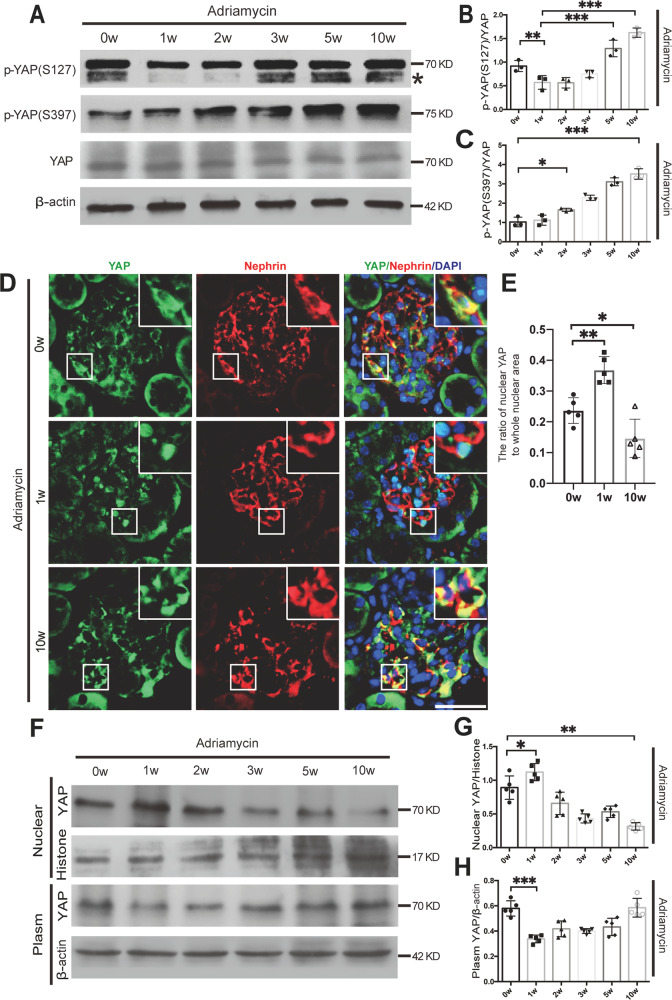
The phosphorylation and distribution of YAP in the Adriamycin-induced FSGS mouse model. **A**–**C** Immunoblotting showed p-YAP (S127) was increased by 5w and p-YAP (S397) was increased by 2 weeks of Adriamycin treatment. **D** Double immunofluorescence staining with YAP and Nephrin revealed relatively uniform distribution of YAP in podocytes nuclear and cytoplasm at 0w, an obvious nuclear aggregation at 1 week and translocating to cytoplasm at 10w in mice with Adriamycin treatment. Shown are representative images from at least five mice with similar results. Images of higher magnification are shown in the right upper corner. **E** Quantification of YAP positive nuclear area in podocytes of each group as described in Fig. 1C. **F**–**H** Immunoblotting of nuclear or cytoplasm protein from glomeruli protein of mice. Shown are representative blots from at least three separate experiments with similar results. *Denotes a nonspecific band. *P < 0.05, **P < 0.01, ***P < 0.001 compared to 0 week or 1 week after Adriamycin treatment (Scale bar: 20 μm in **D**).

We then examined the phosphorylation and distribution of YAP in an Adriamycin-induced FSGS mouse model. Immunoblotting showed that total YAP was gradually reduced in isolated glomeruli from 1st to 10th week (Fig. 3, A). However, the ratio of p-YAP (S127) to total YAP was increased from the 5th week, although it was declined in the first 3 weeks (Fig. 3A, B). Phospho-YAP (S397) which has been known to correlate with YAP degradation was continuously increased during the progression of FSGS (Fig. 3A, C). Double immunofluorescence staining for YAP and nephrin, a podocyte-specific marker, showed that YAP was distributed both in nucleus and cytoplasm at 0 week, while in the nucleus of podocyte at the 1st week but predominantly in the cytoplasm at the 10th week of Adriamycin treatment (Fig. 3D, E). In addition, cell nuclear and cytoplasm protein extracted from the isolated glomeruli were subjected to immunoblotting analysis. As shown in Fig. 3F–H, the protein expression of nuclear YAP was increased in the first week after Adriamycin treatment followed by gradually reduction, but the cytoplasmic YAP expression was decreased in the first week followed by gradually upregulation. These results suggest that Adriamycin-induced gradual nuclear exclusion of YAP in podocyte.

**Fig. 4:**
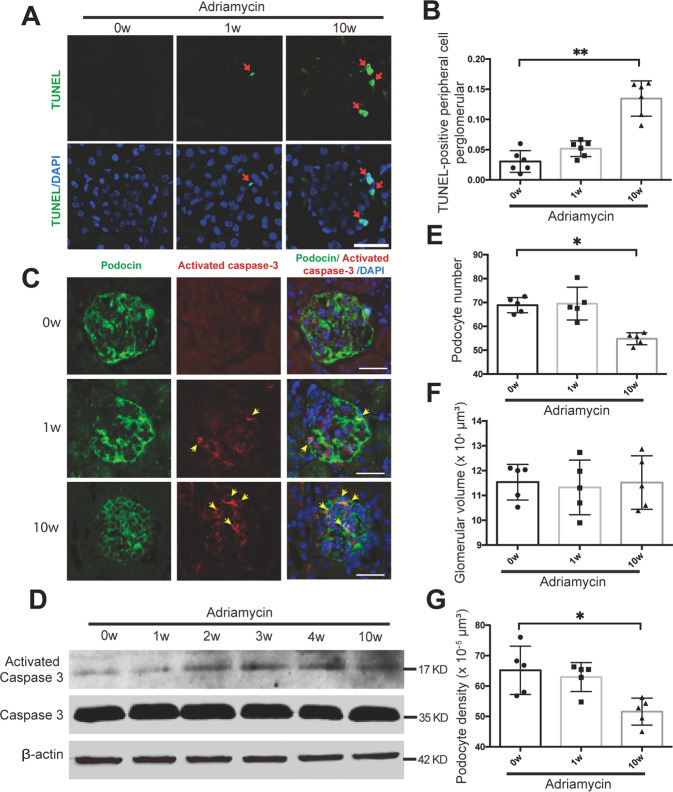
Adriamycin treatment induced podocyte apoptosis in the progressive stage of FSGS. **A** TUNEL staining was used to label apoptotic cell and DAPI to label nuclei, showing markedly increased apoptotic cells in peripheral area of glomeruli in mice sacrificed at 10th week after Adriamycin treatment compared to other groups. **B** Apoptotic cells in peripheral area of glomeruli were calculated from 25 randomly captured images at each time point (5 images per mouse, 5 mice per group). Only TUNEL-positive nuclei in peripheral area of glomeruli was counted for the numerator, which was divided by the sum of DAPI-positive nuclei and TUNEL-positive nuclei within glomeruli. **C** Double immunofluorescence staining with activated Caspase 3 and podocyte cytoplasm marker podocin. Representative apoptotic podocytes were marked with arrow (yellow). **D** Immunoblotting of glomeruli protein showed upregulated protein expression of activated Caspase 3 with the progression of Adriamycin-induced FSGS. Shown are representative blots from at least three separate experiments with similar results. **E** Podocyte number was measured and calculated using the dissector/fractionator principle that nuclei present in the first section of each dissector pair but not in the second section were counted. **F** Glomerular volume was not change significantly during FSGS progression, measured by the Cavalieri principle. **G** Podocyte density was calculated by dividing podocyte number by glomerular volume (*n* = 5 mice, 50 total glomeruli per group). Data are expressed as means ± SEM, *P < 0.05, **P < 0.01 (Scale bar: 20 μm in **A**, **C**).

Subsequently, we examined podocyte apoptosis in the progression of FSGS. TUNEL assay showed that TUNEL-positive podocyte was increased after Adriamycin treatment (Fig. 4A, B). Double immunofluorescence staining of activated Caspase 3 and podocyte cytoplasm marker podocin further confirmed the increase of apoptosis in podocytes (Fig. 4C). Immunoblotting of isolated glomerulus proteins showed the protein expression of active Caspase 3 was increased from the 2nd week of FSGS mouse model (Fig. 4D). We further quantified podocyte number, glomerular volume and podocyte density in an Adriamycin-induced FSGS mouse model (*n* = 5 in each group), and found the number of podocytes per glomeruli and podocyte density were decreased at the 10th week of Adriamycin treatment without change of glomerular volume (Fig. 4E–G; Supplementary Fig. 4). These results suggest that gradual nuclear exclusion of YAP was positive correlated to podocyte apoptosis, which contributes to the podocyte loss in the progression of FSGS.

### Verteporfin decreased YAP–TEAD combination and stimulated nuclear exclusion of YAP in primary cultured podocytes

In the primary cultured mG-labeled podocytes, total YAP was also reduced in response to Adriamycin, but the ratio of p-YAP (S127)/YAP was upregulated as was the active Caspase 3 (Supplementary Fig. 2A, B). Notably, the ratio of p-YAP (S127)/YAP was reduced after 12 h of Adriamycin treatment. Immunofluorescence staining of YAP showed that it was located in both cytoplasm and nucleus at 0 h but aggregated in the nucleus at 12 h and in the cytoplasm at 48 h in Adriamycin-treated primary podocytes (Supplementary Fig. 2C).

**Fig. 5:**
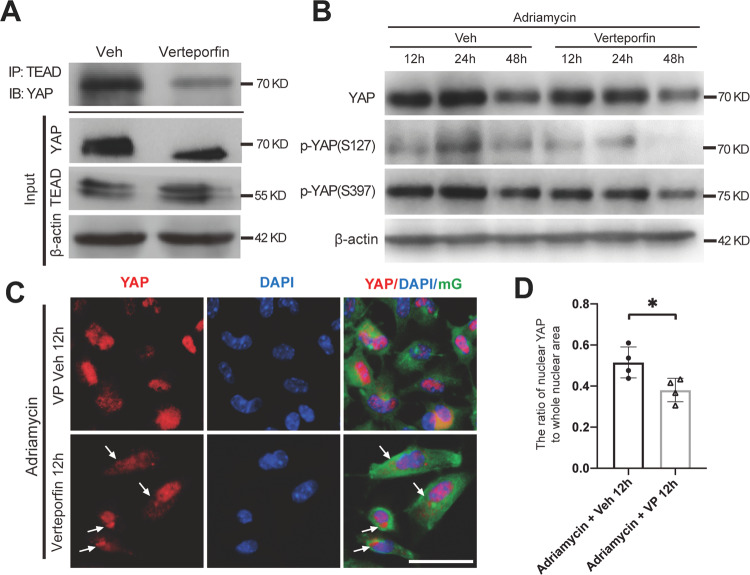
The phosphorylation and distribution of YAP after verteporfin treatment in vitro. **A** Primary podocyte was exposed to verteporfin 250 nmol/L or vehicle for 12 h, and cell lysates were used for immunoprecipitation with antibodies against TEAD, followed by immunoblots with antibodies against YAP. Input was used as a control. **B** Primary podocytes were exposed to 0.25 μg/mL Adriamycin with or without verteporfin concurrently, followed by analysis with immunoblotting. **C** Immunofluorescence staining with YAP in cultured mG-labeled primary podocyte. Arrows point out increased YAP cytoplasm staining in podocytes after 12 h of verteporfin treatment. **D** YAP positive nuclear area was calculated from 20 randomly captured images for each experiment. Only area of YAP and DAPI double-positive staining that surrounded by green mG (indicate the YAP in podocyte nuclear) were counted for the numerator, which was divided by the sum of the area with DAPI that also surrounded by green mG (indicate all the area of podocyte nuclear). Shown are representative immunoblots and immunofluorescence staining images from at least three independent experiments with similar results. Data are expressed as means ± SEM, *P < 0.05 (Scale bar: 25 μm in **C**).

Since the effect of YAP requires binding to TEAD after it enters the nucleus, we used verteporfin to treat podocytes, which reduced the YAP activity by inhibiting its association with TEAD [19, 31], thus changed the nucleus distribution of YAP. We first confirmed that verteporfin treatment blocked YAP–TEAD combination in cultured primary podocytes by using co-immunoprecipitation as shown in Fig. 5A. Then decreased total YAP, p-YAP (S127), and p-YAP (S397), was observed by verteporfin treatment (Fig. 5B). Immunofluorescence staining showed that compared to vehicle control, some YAP began to translocate from nucleus to cytoplasm at 12 h post-verteporfin combined with Adriamycin treatment (Fig. 5C, D). These results indicate that decrease of YAP–TEAD combination in podocyte nuclear lead to earlier and more nuclear exclusion of YAP.

### Verteporfin accelerated FSGS progression by nuclear exclusion of YAP

**Fig. 6:**
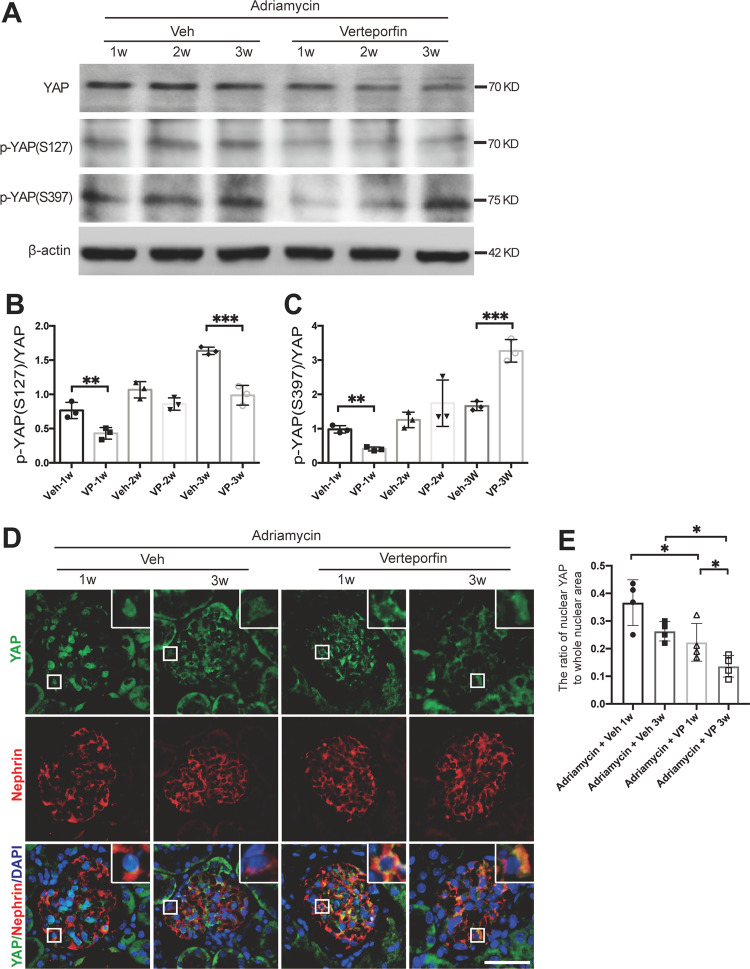
verteporfin treatment induced the nuclear exclusion of YAP in vivo. **A**–**C** At the 1st day of Adriamycin-induced nephropathy mouse model construction, verteporfin (100 mg/kg body wt by intraperitoneal injection once every other day) was given for 1, 2, or 3 weeks. Immnunoblotting of glomeruli protein showed verteporfin significantly decreased total YAP and p-YAP (S127), but increased p-YAP (S397) by 3 weeks of treatment, compared with vehicle control. **D** Double immunofluorescence staining with YAP and nephrin in verteporfin or vehicle treated Adriamycin-induced FSGS mouse model. **E** Quantification of YAP positive nuclear area in podocytes of each group (8 images per mouse, 5 mice per group) as described in Fig. 1C. Data are expressed as means ± SEM. *P < 0.05*,* **P < 0.01*,* ***P < 0.001 (Scale bar: 20 μm).

To determine the effect of verteporfin on the Adriamycin-induced FSGS mouse model, verteporfin was administrated to the mice for 1, 2, or 3 weeks starting at the 1st day of Adriamycin injection. The protein expression of total YAP, p-YAP (S127) and p-YAP (S397) in glomeruli was inhibited and the ratio of p-YAP (S127) to YAP was reduced by verteporfin treatment (Fig. 6A, B). However, the ratio of p-YAP (S397) to YAP was increased at the 3rd week of verteporfin treatment (Fig. 6A, C). Immunofluorescence staining showed that as early as the 1st week after verteporfin treatment, YAP was transferred from the nuclei to the cytoplasm, but was found in podocyte nuclei in vehicle controls. And at the 3rd week, YAP was no longer found in the nucleus and was accompanied by decreased protein expression (Fig. 6D, E). The results suggest that verteporfin induced the translocation of YAP from podocyte nuclear to cytoplasm in vivo.

**Fig. 7:**
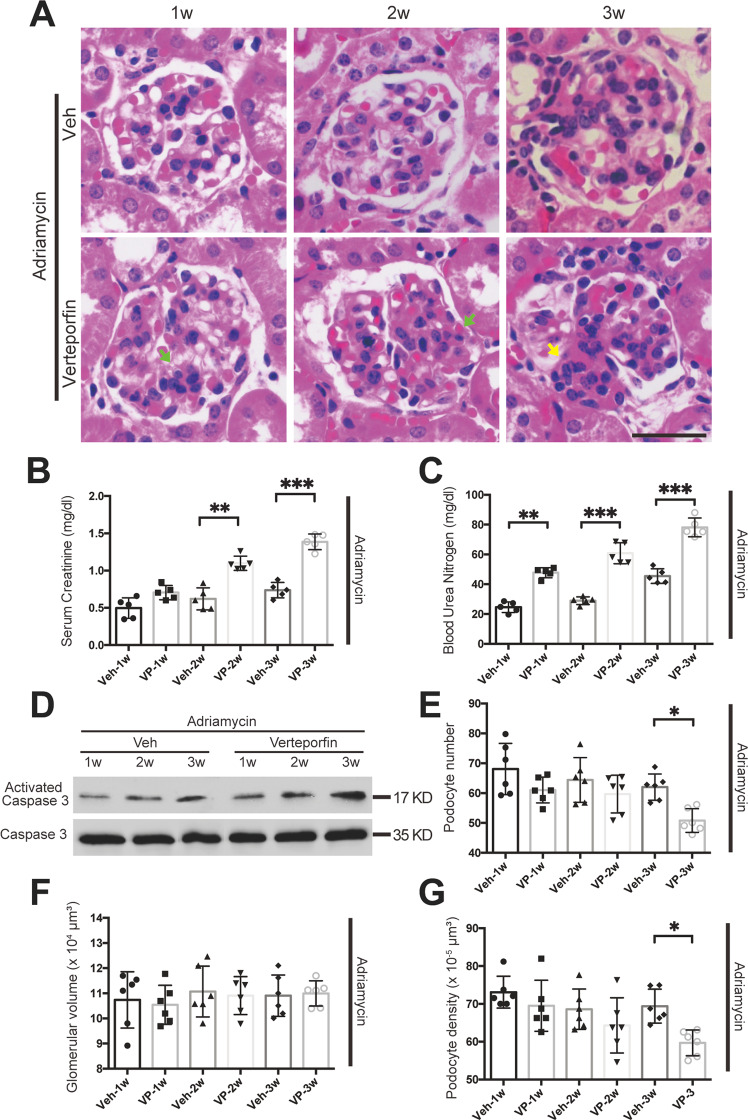
Verteporfin accelerated disease progression in the Adriamycin-induced FSGS mouse model. **A** H&E staining indicate matrix deposition occurring early at 2 weeks and typical segmental glomerulosclerosis at 3 weeks of verteporfin treatment in an Adriamycin-induced FSGS mice. **B**, **C** Measurements of serum creatinine and blood urea nitrogen revealing an accelerating deterioration of kidney function in verteporfin group compared with the vehicle group. **D** Immunoblotting showed upregulated protein expression of activated Caspase 3 after verteporfin administration. **E** Podocyte number was reduced significantly at 3 weeks of verteporfin treatment. **F** Glomerular volume was not change significantly after verteporfin treatment. **G** Podocyte density was reduced at 3 weeks of verteporfin group compared with vehicle group (*n* = 5 mice, 30 total glomeruli per group). Data are expressed as means ± SEM. *P < 0.05, **P < 0.01, ***P < 0.001 (Scale bar: 25 μm in **A**).

We then examined the morphological change of kidney and renal function. As shown in Fig. 7A, little mesangial matrix was observed at the 3rd week of vehicle control. However, extracellular matrix accumulated in the mesangial area by the 1st and 2nd week of verteporfin treatment. Moreover, segmental sclerosis could be found at the 3rd week of verteporfin treatment, which was much earlier than in the vehicle control. In addition, verteporfin elevated the level of serum creatinine and blood urea nitrogen in the FSGS mouse model. Immunoblotting showed that verteporfin increased expression of active caspase 3 (Fig. 7D), significantly reduced podocyte number and density, but did not change the glomerular volume (Fig. 7E–G). These results suggest that verteporfin induced podocyte apoptosis and accelerated the progression of FSGS by inhibiting the formation of YAP–TEAD and stimulating nuclear exclusion of YAP in vivo.

## Discussion

**Fig. 8:**
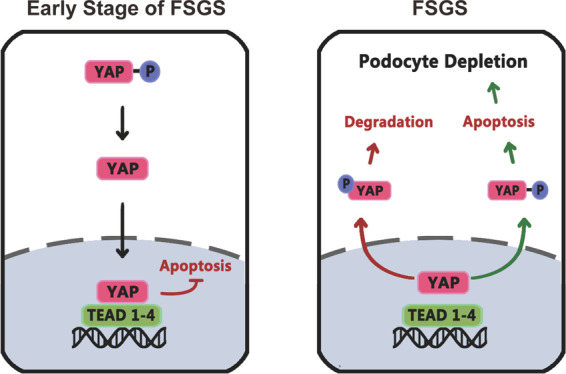
Proposed changes in YAP in podocytes of mice with Adriamycin-induced FSGS. In the early stages of FSGS, YAP mainly accumulated in nucleus of podocytes showing an important role of anti-apoptosis (Left). However, with the progression of FSGS, YAP gradually translocated from nucleus to cytoplasm with significantly enhanced YAP phosphorylation, and causing decrease in antagonizing apoptosis and increase in podocyte depletion. In addition, increased degradation of YAP contributes to lower YAP expression.

Whether podocyte injury leads to YAP nuclear exclusion, or YAP nuclear exclusion promotes podocyte injury in vivo, is still a problem to be explored [32]. In the present study, we found accumulation of nuclear YAP in podocytes in MCD with little podocyte apoptosis. However, in FSGS, a large amount of YAP translocated from nucleus to cytoplasm with significantly enhanced YAP phosphorylation, podocyte apoptosis and loss both in glomeruli with or without segmental glomerulosclerosis. In addition, inhibiting YAP by verteporfin induced YAP’s translocation to cytoplasm, thus increased podocyte apoptosis and accelerated the disease progression in the Adriamycin-induced FSGS model. Therefore, YAP nuclear exclusion is the driving force for podocyte injury (Fig. 8).

YAP is regulated by multiple mechanisms, such as cell–cell contact [9, 33, 34], cell polarity [35, 36], and actin cytoskeleton [37], as well as some other signals, including cellular energy status [38], mechanical stimulates [39], and hormonal signals that act through G-protein-coupled receptors [40]. What we used to induce FSGS in the present study is Adriamycin, a well-known inducer of renal injury in rodents to mimic the histological changes of human glomerulosclerosis [41], inducing effacement of podocyte foot processes (Fig. 2B) and diminution of glomerular endothelial surface layer [42]. The effacement of podocyte foot process leads to a decrease of podocyte tight junction and cell density, which evokes the activation of Hippo pathway and subsequently increase of both p-YAP (S127) and p-YAP (S397) in angiomotin-dependent manner [43]. Thus, we observed a gradual and continuous nuclear exclusion in advanced stage of the FSGS mouse model, which accompanied by apoptosis and loss of podocytes, and decreased renal function. It is worth noting that in the first week after ADR injection, YAP accumulated in the nucleus, at which time podocytes showed no apoptosis, as well as changes in podocyte number and renal function. We think that at this stage, YAP entering the nucleus is just a transient protective response to ADR stimulation.

Verteporfin, functioning via a reactive oxygen-dependent mechanism, was considered as a kind of small molecule for treatment of ocular photodynamic [44]. Recent studies showed verteporfin could inhibit YAP activity [20, 45], by interfering with its interaction with the TEAD [46]. Here we found verteporfin not only inhibited the interaction between YAP and TEAD, but also reduced total YAP. Reduction of YAP synthesis or direct enhancement of protein aggregation through oxidative crosslinking induced by verteporfin may contribute to total YAP decrease, but the detailed underlying mechanism needs more investigation [47, 48]. Szeto et al. showed that treatment of verteporfin increased Smad accumulation and attenuated renal fibrosis by reduced YAP/TAZ level [49]. However, in our study, verteporfin inhibited YAP–TEAD combination, which promoted the sustained nuclear exclusion of YAP in podocytes, so that podocyte apoptosis and FSGS progression were accelerated.

Schwartzman et al. observed that glomerular YAP expression was reduced, similar to the distribution of synaptopodin in human primary FSGS [16]. We also found decreased YAP expression in human FSGS, an Adriamycin-induced FSGS mouse model and Adriamycin-treated podocytes. Meanwhile, we found a gradually enhanced phosphorylation of YAP on Serine 397, which lead YAP for following phosphorylation through casein kinase 1δ/ε (CK1 δ/ε) in a phosphodegron, resulting in recruitment of E3-ubiquitin ligase β-transducin repeat-containing proteins and following degradation of YAP via ubiquitin-proteasome pathway [50]. Therefore, p-YAP(S397) may contribute to total YAP reduction in FSGS. Another phosphorylated site of YAP, S127, was found transiently decreased and then increased steadily in response to Adriamycin treatment in our study. Phospho-YAP(S127) is important to sequestrate YAP in cytoplasm and then bind with 14-3-3 domain [9], so it is a good indicator of YAP distribution. Decreased p-YAP (S127) indicates a reduction of cytoplasmic YAP, whereas increased p-YAP(S127) indicates an increase distribution of cytoplasmic YAP. Therefore, increase of p-YAP (S127) in advanced stage of FSGS contributed to the nuclear exclusion of YAP. In addition, we observed that the changes of YAP of podocyte was the same both in glomeruli with or without lesions at the same stage, since although FSGS is a focal segmental lesion, podocyte injury is thought to be a diffuse phenomenon.

In summary, our study suggests that YAP activation and nuclear distribution in podocytes is an important endogenous anti-apoptotic mechanism during the progression of FSGS and targeting YAP could be a potential therapy for treatment of FSGS.

## Supplementary information

Supplemental material
