# Supplementary material for: Nuclear exclusion of YAP exacerbates podocyte apoptosis and disease progression in Adriamycin-induced focal segmental glomerulosclerosis
Source: Lab Invest. 2020 Nov 17;101(2):258–70. doi: 10.1038/s41374-020-00503-3 (PMC7815513; doi:10.1038/s41374-020-00503-3)
Supplement: Supplementary file 1 — Supplemental material [file 41374_2020_503_MOESM1_ESM.pdf]

Supplementary Information

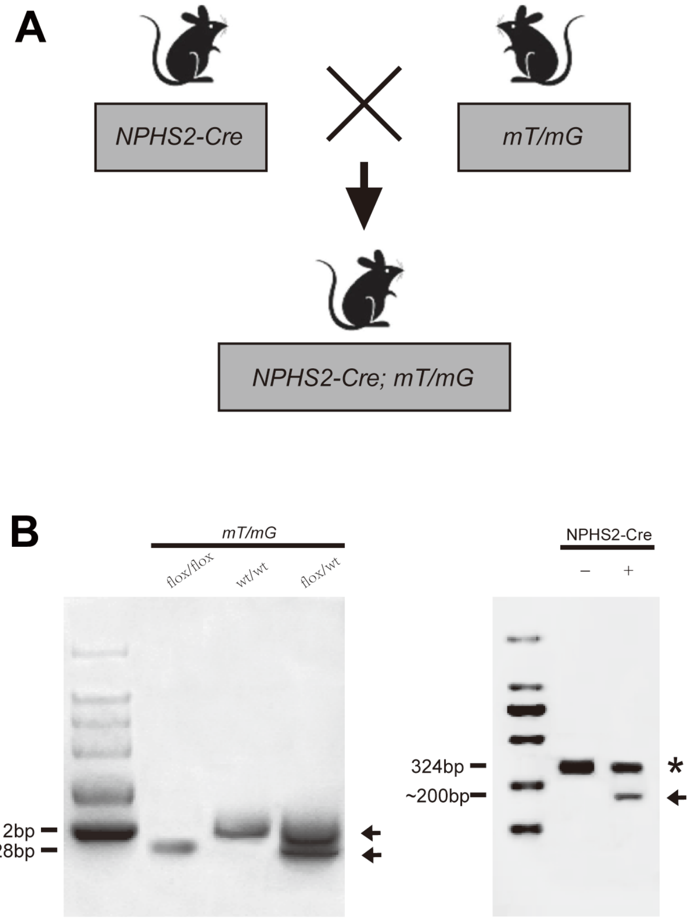

Supplementary Figure 1. Generation of NPHS2 Cre; mT/mG mice. (A) Strategy for making mT/mG mice expressing GFP specifically in podocytes. (B) Identification of mT/mG mice and NPHS2-Cre mice by PCR. *Denotes an internal positive control.

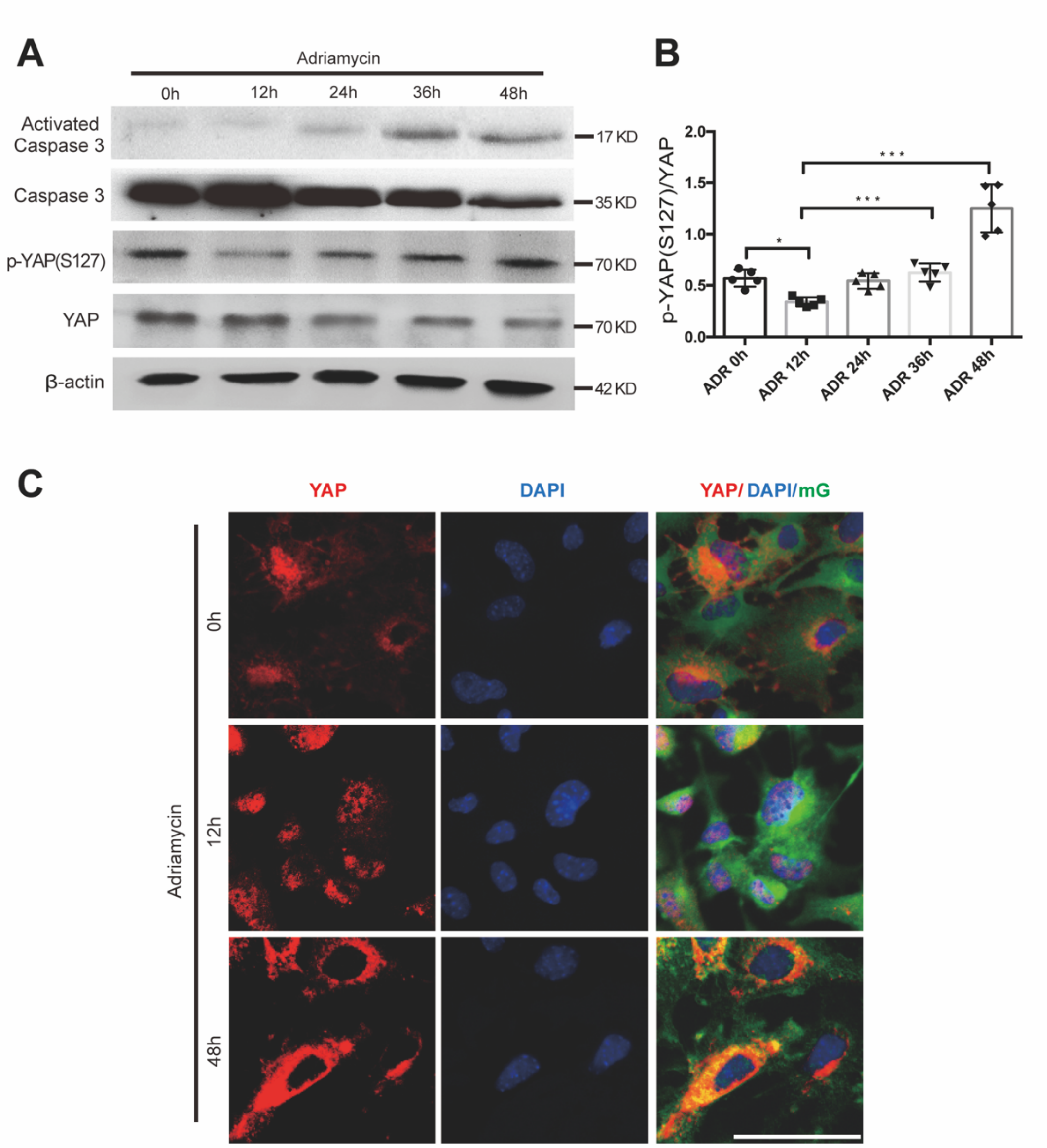

Supplementary Figure 2. Expression and distribution of YAP in primary podocyte treated with Adriamycin. (A-B) Cultured primary podocyte was exposed to 0.25μg/ml ADR for 0, 12, 24, 36 or 48h, followed by analysis with immunoblotting. **P <0.05, ***P <0.001*. (C) Immunofluorescence staining with YAP in cultured mG-labeled primary podocyte demonstrating a massive nuclear distribution at 12h after Adriamycin treatment and then an obvious translocation from nuclear to cytoplasm at 48h. Shown are representative immunoblots and immunofluorescence staining images from at least three independent experiments with similar results. (Scale bar: 25μm)

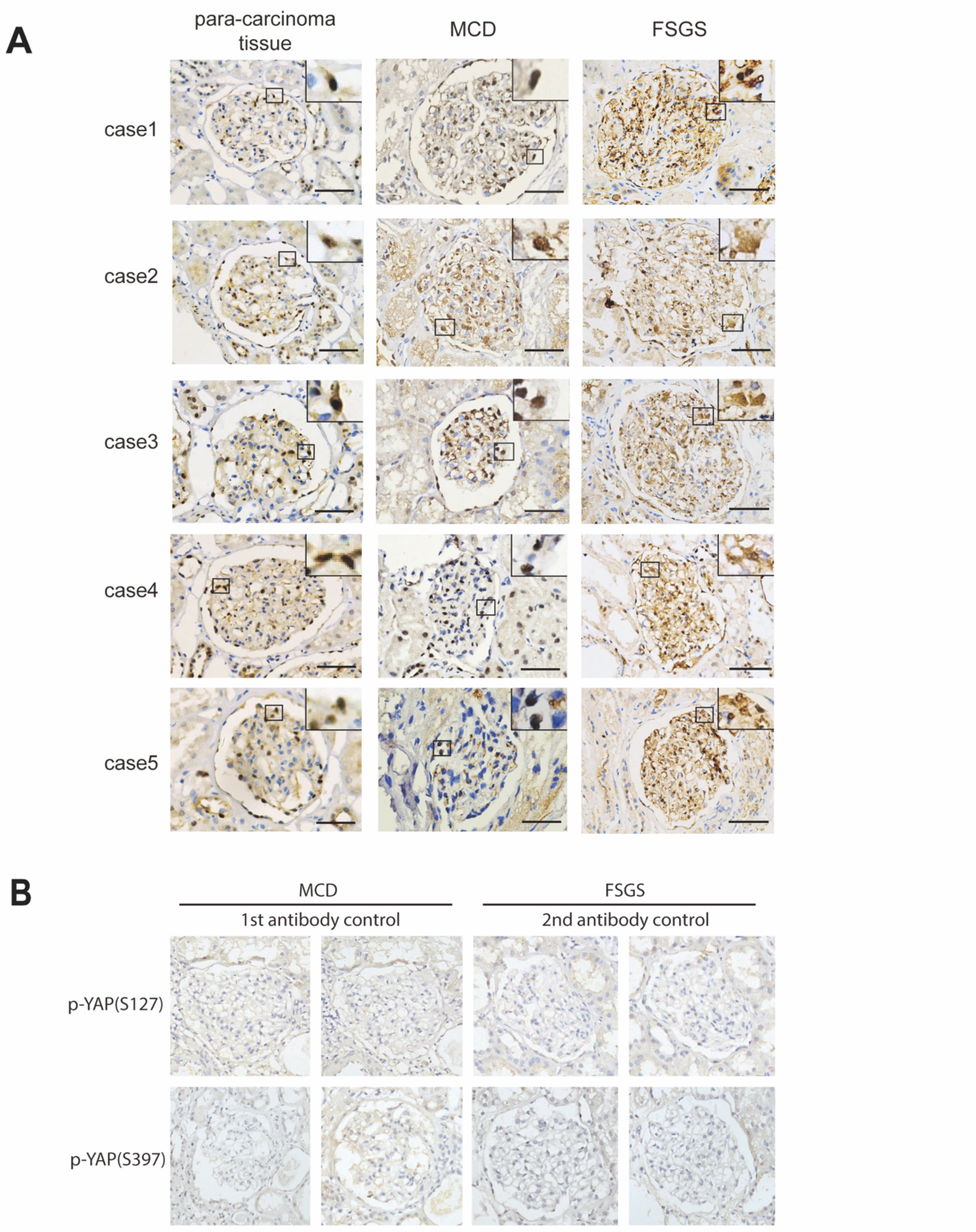

Supplementary Figure 3. YAP expression in cases of para-carcinoma tissue, MCD and FSGS. (A) Shown are representative images of immunohistochemical staining with YAP from para-carcinoma tissue, MCD and FSGS cases (n=5 for each group). (B) The 1st and the 2nd antibody controls for p-YAP (S127) and p-YAP (S397). (Scale bar: 20μm)

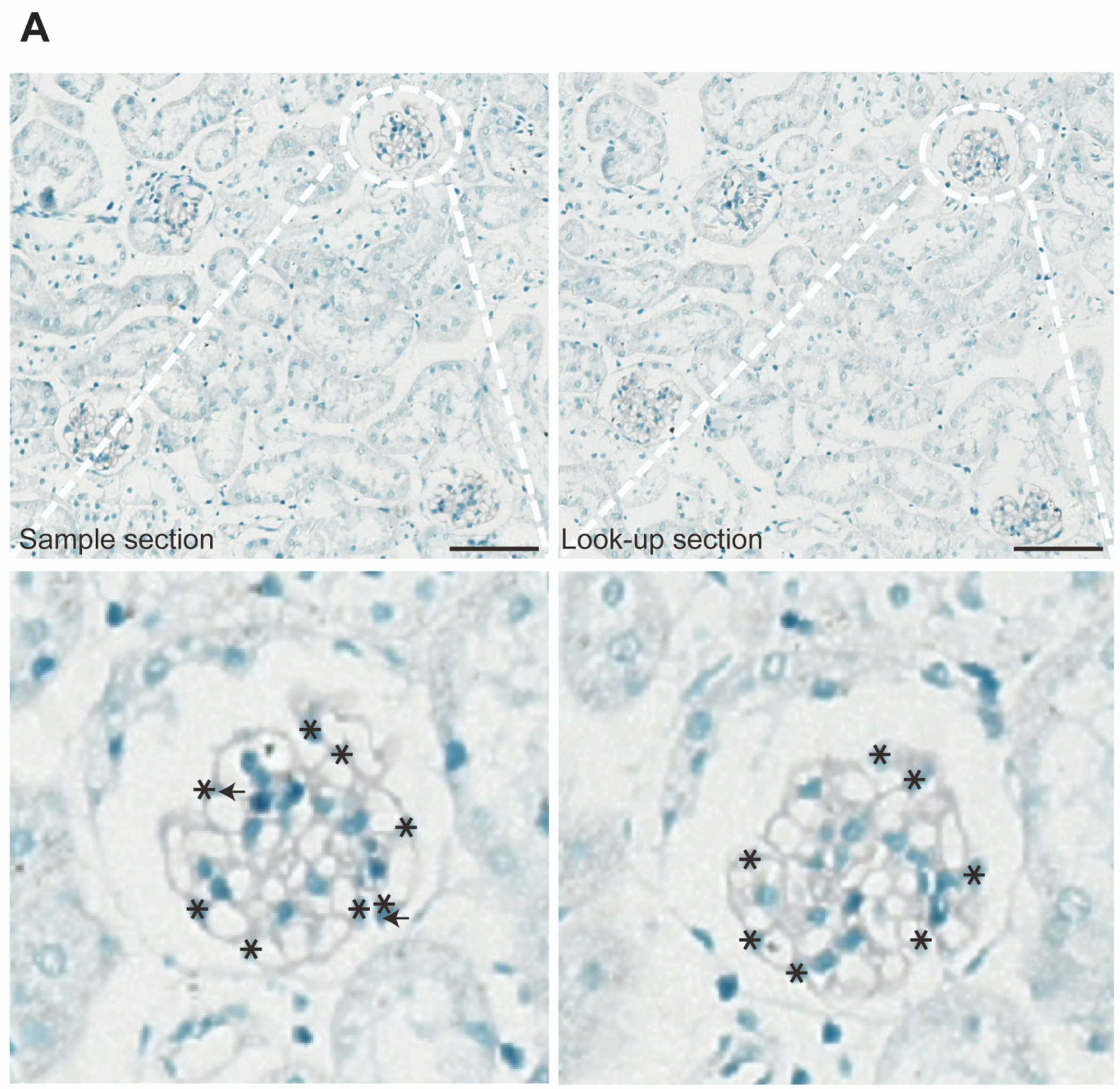

Supplementary Figure 4. Toluidine-blue staining of the kidney tissue for quantification of podocyte number. (A) Toluidine-blue staining showed quantification of podocyte number in adjacent sections with 2 µm thick. The unique nuclei (asterisk) only present in the first section of each dissector pair but not in the look-up (arrows) were counted. (Scale bar: 20µm)
